# Steroid‐Refractory Cryptogenic Organising Pneumonia (COP) in a Patient With Mannose‐Binding Lectin (MBL) Deficiency

**DOI:** 10.1002/rcr2.70494

**Published:** 2026-01-29

**Authors:** Ilias E. Dimeas, Sotirios I. Sinis, Paraskevi Kirgou, Cormac McCarthy, Zoe Daniil

**Affiliations:** ^1^ School of Medicine University College Dublin Dublin 4 Ireland; ^2^ Department of Respiratory Medicine St. Vincent's University Hospital Dublin 4 Ireland; ^3^ Department of Respiratory Medicine, Faculty of Medicine University of Thessaly Larissa Greece

**Keywords:** cryptogenic organising pneumonia, hydroxychloroquine, immunomodulation, mannose‐binding lectin deficiency, steroid‐refractory disease

## Abstract

We report a 67‐year‐old man with a relapse characterised by fever, respiratory failure and bilateral infiltrates following completion of a short‐term empirical methylprednisolone regimen for a similar episode. Serum, upper respiratory tract and bronchoalveolar lavage samples were negative for infection, whereas an extensive antibody panel showed no remarkable findings. The presence of migratory opacities on chest imaging, a mixed cellular lavage pattern and prior steroid responsiveness supported a provisional diagnosis of cryptogenic organising pneumonia. During outpatient follow‐up, frequent exacerbations occurred, barring any steroid tapering attempts. An individualised pathophysiological hypothesis is proposed for the recalcitrant course following incidental detection of mannose‐binding lectin deficiency. Given the potential role of mannose binding lectin in apoptotic cell clearance and modulation of inflammation, we postulate an impingement on the disease trajectory, which has been previously observed in chronic obstructive pulmonary disease, bronchiectasis and bronchiolitis obliterans post‐transplant.

## Graphical abstract

A 67‐year‐old man with cryptogenic organising pneumonia developed recurrent relapses despite corticosteroid and mycophenolate therapy. A complete absence of mannose‐binding lectin was identified, suggesting impaired apoptotic cell clearance as a contributor to persistent inflammation. Disease remission was finally achieved with low‐dose hydroxychloroquine, highlighting a potential immunomodulatory approach in steroid‐refractory cases.

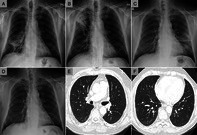

## 1 Introduction

Cryptogenic organising pneumonia (COP) is a rare form of idiopathic interstitial pneumonia presenting with variable high resolution computed tomography (HRCT) patterns. The pathogenesis involves a lung injury response of unknown cause, producing the characteristic histopathological pattern of organised fibrous exudate within distal airspaces. The condition is termed secondary organising when a predisposing factor is identified, such as infection, toxic substance exposure, medication, connective tissue disease, malignancy, transplantation and radiotherapy. Exclusion of alternative diagnoses, particularly in the absence of adequate biopsy specimens, remains essential. COP usually follows a subacute course and responds to corticosteroids, though relapses are common. Severe hypoxemia, delayed therapy, residual lesions and fibrotic radiological features predict refractory disease [1].

Mannose‐binding lectin (MBL) is a soluble innate immune molecule that recognises mannose, fucose and N‐acetylglucosamine residues on many pathogens. By activating the lectin complement pathway, MBL enhances phagocytosis and leukocyte recruitment but also dampens lung inflammation by promoting apoptotic cell clearance through efferocytosis [2, 3]. MBL deficiency is relatively common genetically, but clinically significant deficiency is uncommon. It may increase susceptibility to infection and autoimmune disease and has been associated with poorer outcomes in cystic fibrosis, bronchiectasis, chronic obstructive lung disease and post‐transplant settings [3, 4]. However, no studies have investigated the course of COP in the presence of MBL deficiency.

## 2 Case Report

A 67‐year‐old male with a 50 pack‐year smoking history was referred to our tertiary center for a relapse characterised by fever, nocturnal sweating, respiratory failure and bilateral infiltrates occurring after completion of a short empirical corticosteroid regimen that followed initial antibiotic therapy. The illness began 60 days before presentation with fever and nocturnal sweats. The patient remained homebound for 15 days, was then hospitalised at a regional hospital, and discharged 1 month prior to referral. According to available records, the condition was refractory to intravenous broad‐spectrum antibiotics and improved partially after corticosteroids initiation, although long‐term oxygen therapy was still required at home.

**FIGURE 1:**
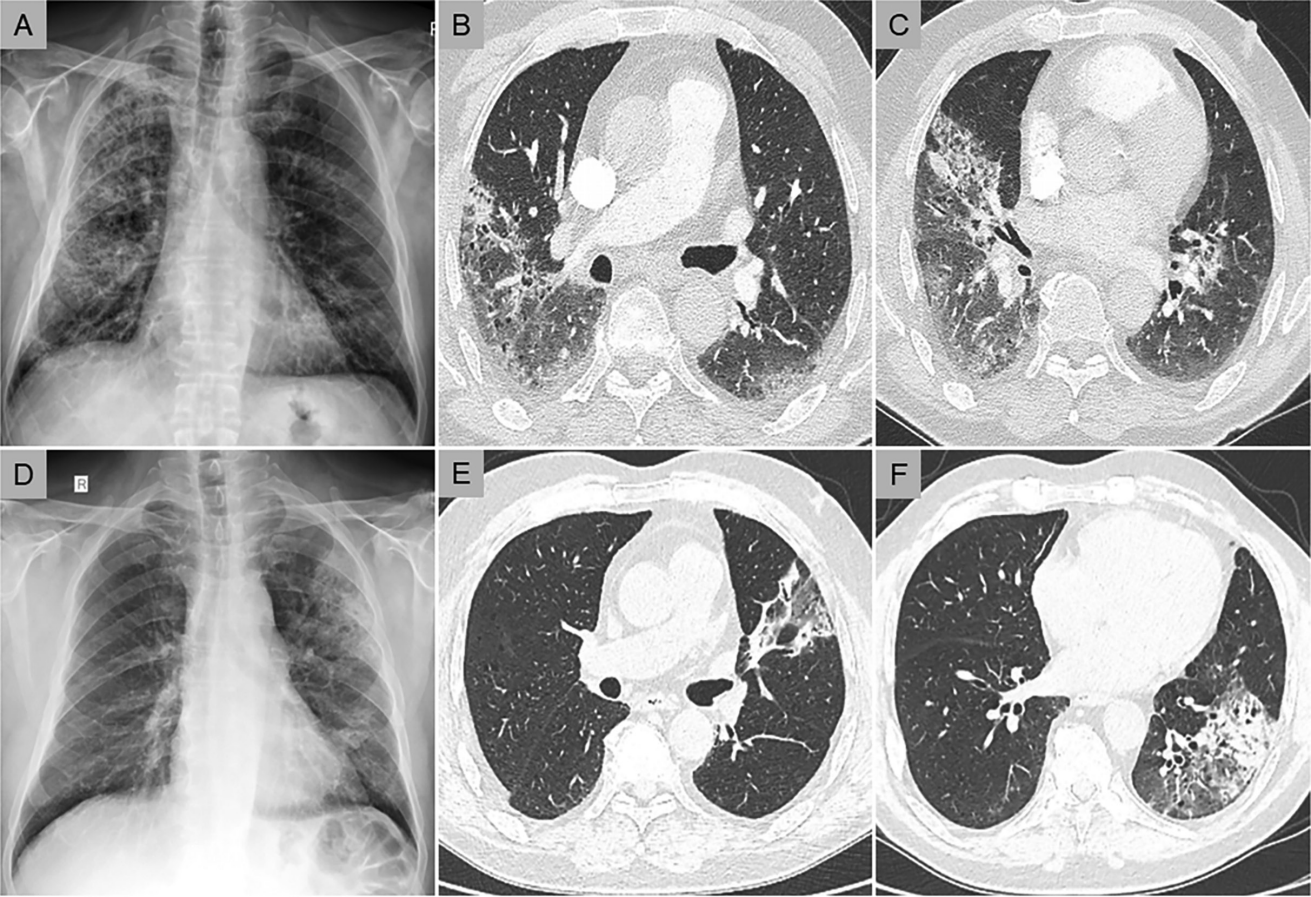
Radiological findings showing a migratory pattern of pulmonary infiltrates. (A) Admission chest radiograph demonstrating bilateral alveolar infiltrates, more prominent on the right, with subtle interstitial prominence. (B, C) High‐resolution computed tomography images at presentation revealing bilateral peripheral consolidations and ground‐glass opacities. (D–F) Earlier imaging showing pulmonary infiltrates that later resolved, whereas new lesions subsequently developed in different lung regions, consistent with a migratory pattern.

Upon admission, he was febrile, with oxygen saturation of 85% at ambient air and a respiratory rate of 28/min. He required 50% FiO_2_ to maintain adequate oxygenation. Fine bilateral crackles were evident predominantly at the lower lung fields; no lymphadenopathy was palpated. Laboratory testing showed elevated C‐reactive protein and erythrocyte sedimentation rate with normocytic normochromic anaemia; tumour markers were normal. The admission chest radiograph (Figure 1A) demonstrates bilateral alveolar infiltrates, more prominent on the right, with subtle interstitial prominence. An extensive autoantibody panel, including rheumatoid factor, antinuclear antibodies, anti‐cyclic citrullinated peptide antibodies, perinuclear and cytoplasmic anti‐neutrophil cytoplasmic antibodies, as well as antibodies associated with antisynthetase syndrome, dermatomyositis, immune‐mediated necrotising myopathy, and overlap myositis, was unremarkable. No infection was detected by serology; by sputum, blood and bronchoalveolar lavage (BAL) cultures; or by PCR on upper respiratory tract and BAL samples. BAL immunophenotyping showed a mixed cellular pattern. Chest HRCT (Figure 1B,C) revealed bilateral peripheral consolidations and ground‐glass opacities, predominantly in the lower lobes. Comparison with prior imaging (Figure 1D–F) confirmed a migratory pattern. Given prolonged steroid exposure and concurrent normocytic anaemia, contrast‐enhanced abdominal CT and gastroscopy were performed; the only notable finding was fungal oesophagitis, treated with topical oral antifungal therapy. The diagnostic work‐up is summarised in Table 1.

**TABLE 1:** Summary of investigations performed during the diagnostic work‐up.

Diagnostic domain	Investigation	Result
Connective tissue disease/autoimmunity	Antinuclear antibodies	Negative
	Rheumatoid factor	Negative
	Anti‐cyclic citrullinated peptide	Negative
Vasculitis	Perinuclear and Cytoplasmic anti‐neutrophil cytoplasmic antibodies	Negative
Idiopathic inflammatory myopathies	Antisynthetase antibodies	Negative
	Dermatomyositis‐specific antibodies	Negative
	Immune‐mediated necrotising myopathy antibodies	Negative
	Overlap myositis antibodies	Negative
Infection	Blood, sputum, BAL cultures	Negative
	Upper and lower respiratory PCR panels	Negative
Malignancy	BAL cytology	Negative for malignancy
Hypersensitivity pneumonitis	Serum precipitins	Negative
	Exposure history	No relevant exposure identified
Chronic eosinophilic pneumonia	BAL differential cell count	No eosinophilia
Cryoglobulinemia	Serum cryoglobulins	Negative
Humoral immunity	Serum Immunoglobulin G	Mild hypogammaglobulinemia
Cellular immunity	Clinical history of opportunistic viral or fungal recurrent infections	Absent
Phagocyte function	Complete blood count with differential	Normal neutrophil count
Complement/innate immunity	Serum mannose‐binding lectin	Markedly reduced
	Repeat serum MBL (after 3 months)	Persistently reduced
Genetic confirmation	MBL2 gene	LXPA/LXPA haplotype

**FIGURE 2:**
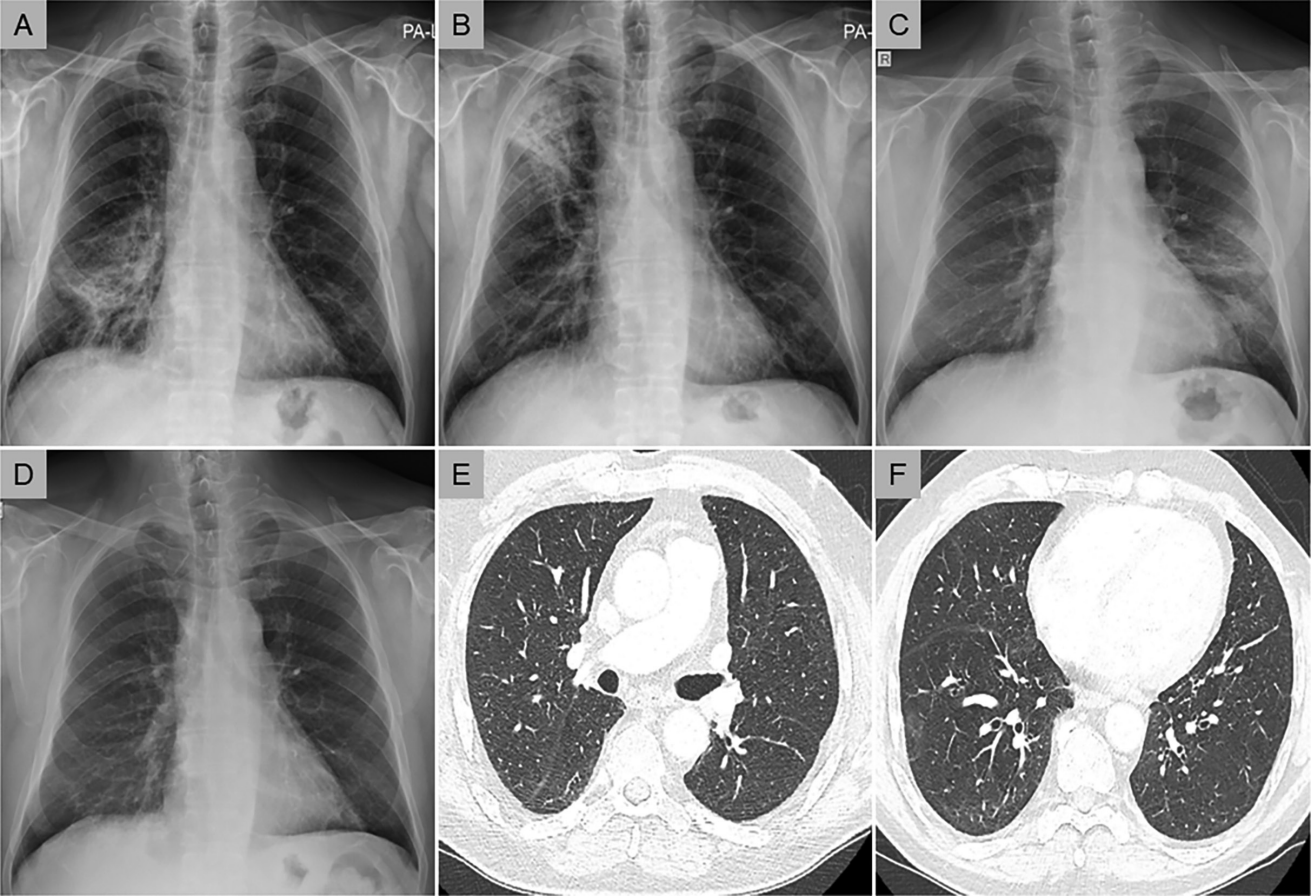
Radiological evolution and treatment response during disease course. (A–C) Serial chest radiographs during outpatient follow‐up showing recurrent migratory patchy infiltrates following attempts at steroid discontinuation. (D–F) Subsequent imaging after introduction of hydroxychloroquine demonstrating sustained resolution of opacities and maintenance of remission.

A provisional diagnosis of cryptogenic organising pneumonia was established based on the subacute steroid‐responsive course; the migratory radiological pattern; and the reasonable exclusion of autoimmune, infectious and malignant disease. Lung biopsy was deferred due to unacceptable risk in the context of clinical deterioration. Complete clinical recovery was achieved at the initial high dose of corticosteroids within 3–5 days, after which a gradual tapering plan was implemented. Over the course of the subsequent follow‐up, multiple attempts at discontinuation failed (Figure 2A–C) with relapses consistently occurring during attempts to taper oral corticosteroids below 7.5 mg prednisolone equivalent. Eventually, steroid‐associated adverse effects necessitated the addition of mycophenolate mofetil, administered for 4 months at doses ranging from 1 to 3 g daily, albeit with minimal benefit.

Incidental discovery of mild hypogammaglobulinemia, most likely due to prolonged (2 years) corticosteroid therapy, was further investigated with a comprehensive immunodeficiency work‐up revealing complete absence of MBL. Diminished serum MBL levels were initially detected and reconfirmed on repeat testing after 3 months to exclude transient changes. Genetic testing using next‐generation sequencing confirmed an MBL2 haplotype associated with complete deficiency (LXPA/LXPA). A comprehensive immunological evaluation excluded other immunodeficiency disorders. Although clinically dormant prior to the present illness, this profound deficiency may acquire pathogenic significance in the context of cryptogenic organising pneumonia and prolonged immunosuppression. In light of these findings, mycophenolate mofetil was discontinued and a trial of low‐dose hydroxychloroquine (200 mg daily) was introduced as a less potent non‐steroidal agent, given the limited response to mycophenolate. This empirical adjustment was followed, within approximately 1 month, by a marked steroid dose reduction, eventual discontinuation and maintenance of clinical and radiological remission (Figure 2D–F).

## 3 Discussion

This case illustrates an unusually recalcitrant form of COP characterised by severe respiratory failure, corticosteroid dependence and frequent relapses despite the addition of mycophenolate mofetil, a suggested steroid‐sparing agent in case series. It is challenging to elucidate whether the course was idiosyncratic or associated with an underlying culprit. A reasonable argument could be formulated for a lung‐confined seronegative autoimmune disease presenting as secondary OP. The aggressive progression and marked improvement after the hydroxychloroquine trial bode well in this regard [1]. As the patient received immunosuppressants throughout follow‐up, extrapulmonary manifestations may have been masked. However, a comprehensive serial rheumatologic evaluation during follow‐up did not support an underlying systemic or lung‐dominant autoimmune disease, and the total absence of clinically evident extrapulmonary features over prolonged follow‐up favoured a diagnosis of cryptogenic rather than secondary organising pneumonia.

One can make a provocative hypothesis implicating MBL deficiency in the present phenotype of COP, as previously described in other lung diseases [2]. Although often redundant under physiological conditions, MBL deficiency during COP may impair debris, apoptotic cell and immune‐complex clearance via reduced complement activation and efferocytosis [2, 4]. In turn, an aberrant resolution of the intra‐alveolar organising process could render the patient susceptible to relapse. Within that context, the benefit of low‐dose hydroxychloroquine deserves further attention. Apart from the non‐specific anti‐inflammatory property, the drug has a propensity to modulate innate immunity through endosomal toll‐like receptor signalling and suppression of macrophage‐derived cytokine release; thus, potentially countering the putative sequelae of MBL deficiency [5]. Although no firm conclusion can be drawn from a single clinical case, it would be worthwhile to explore whether MBL deficiency intersects with COP pathophysiology.

## Author Contributions

Ilias E. Dimeas and Sotirios I. Sinis contributed equally to this work and share joint first authorship. Ilias E. Dimeas conceived the case report, collected clinical data, drafted the manuscript and coordinated the overall project. Sotirios I. Sinis drafted the manuscript and contributed to the literature review. Paraskevi Kirgou assisted in clinical management and data acquisition. Cormac McCarthy contributed to pathophysiological interpretation, critical revision and supervision of the final version. Zoe Daniil provided overall supervision, conceptual guidance and final approval of the manuscript. Authors are affiliated with different institutions in Greece and Ireland as part of an established clinical and research collaboration between the University of Thessaly and University College Dublin on interstitial and rare lung diseases.

## Funding

The authors have nothing to report.

## Ethics Statement

The authors have nothing to report.

## Consent

The authors declare that written informed consent was obtained for the publication of this manuscript and accompanying images using the consent form provided by the Journal.

## Conflicts of Interest

The authors declare no conflicts of interest.

## Data Availability

The data that support the findings of this study are available from the corresponding author upon reasonable request.
